# Supplementary material for: Metasurface enabled broadband all optical edge detection in visible frequencies
Source: Nat Commun. 2023 Oct 14;14:6484. doi: 10.1038/s41467-023-42271-w (PMC10576829; doi:10.1038/s41467-023-42271-w)
Supplement: Supplementary file 1 — Supplementary Information [file 41467_2023_42271_MOESM1_ESM.pdf]

Supplementary Information:

Metasurface enabled Broadband All Optical Edge Detection in Visible Frequencies

Ibrahim Tanriover[1], Sina Abedini Dereshgi[1] and Koray Aydin[1*]

[1]Department of Electrical and Computer Engineering, Northwestern University, Evanston, Illinois 60208, United States

[*] Corresponding author: aydin@northwestern.edu

SI-1 Comparison of 2nd Order Differentiation and High-pass Filtering

We numerically calculated the output of a 4f system with conventional sharp high-pass filters (HPFs) and a second order differentiator as spatial filters. To indicate the differences of the transfer functions apart from the other limitations and to avoid other sources of errors, we calculated system output using fast-fourier transformation. For the calculations, the image size is set to 251x251 pixels.

Two different HPFs are demonstrated as example cases; an opaque disk with 50 pixels diameter and an opaque disk with 200 pixels diameter as seen in Figure S1. Here, we assumed the spatial feature sizes are much larger than the operation wavelength as the required transfer function can be easily obtained using opaque disks in ray optics regime, yet they fail when the diffraction effects take over in smaller dimensions[1]. For the HPFs, the resulting transmission profile becomes a pattern of dark (disk) and bright (everywhere else) regions in spite of our metasurface, which can provide intermediate transmission values due to its sub-wavelength features.

To quantitatively compare the edge detection quality, we calculated the signal-to-noise ratios (SNR) of the output images shown in Figure S2. The SNR is calculated in dBs as,

$$SNR = 10\log_{10}\left(\sum\sum\frac{r(x,y)^2}{[r(x,y)-s(x,y)]^2}\right) \qquad (1)$$

where *s(x,y)* is the output image and *r(x,y)* is only the edges at the output image. The resulted values are 12.8 dB, 14.5 dB, and 26.3 dB, respectively for 50 pixel HPF, 200 pixel HPF, and 2nd order differentiator. The quality difference can also be visually inspected and qualitatively observed from the images and their cross sections (x=0th pixel) provided in Figure S2.

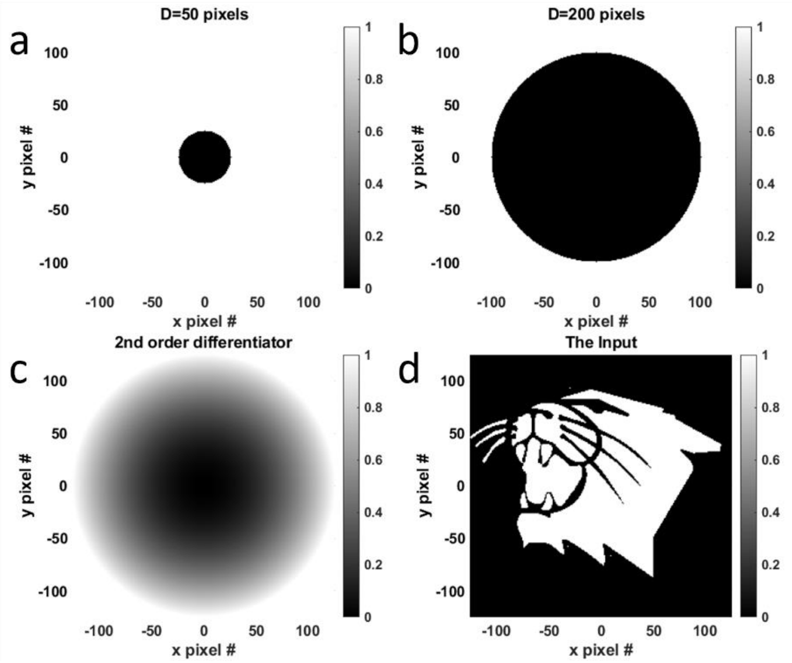

Figure S1. The spatial filters and the input field. The transmission profile of the annular apertures; (a) opaque disk with 50 pixel diameter, (b) opaque disk with 200 pixel diameter, (c) 2nd order differentiator. (d) The input field.

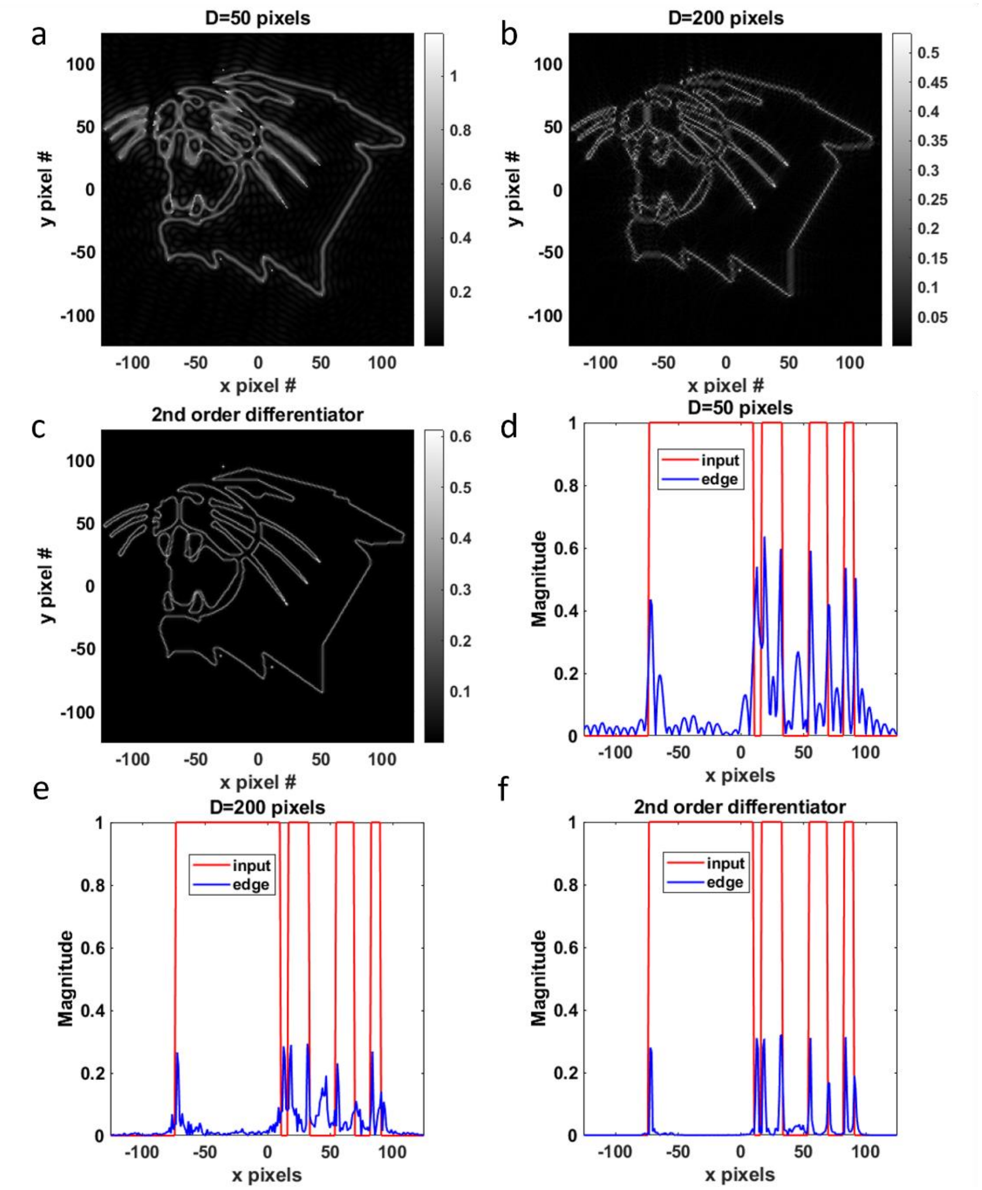

Figure S2. (a-c) The output field of the 4F system corresponding to spatial filter transmission profiles provided in Fig S1 (a-c), respectively. The cross sections along x=0th pixel line for; (d) 50 pixel diameter opaque disk case, (e) 200 pixel diameter opaque disk case, (f) 2nd order differentiator.

SI-2 Unit Cell Simulations in NIR

Figure S3 shows the transmission and phase response of the unit cell library along the near-IR (NIR) range from 800 nm to 1600 nm wavelengths. As seen in the Figure S3 a, all the unit cell library provides the required range of transmission values. Additionally, there is no resonances disrupting the spectral response, which results in gradual and smooth differences between the frequency points enabling broadband operation. Figures S1c-f shows the comparison of optical response of the selected unit cell set and the target response. As seen in the figure, the selected set of unit cells block the smaller spatial frequency components, which is essential for edge detection. The created set also provides the required gradual increase in transmission and phase deviations within a small range. However, the unit cell transmission is larger than the target response as their corresponding position moves away from the center, i.e. towards larger spatial frequencies, which will be an error sources for the images with large spatial frequencies but mainly tolerable. Also note that the difference between the unit cells and the target response increases with the operation wavelength.

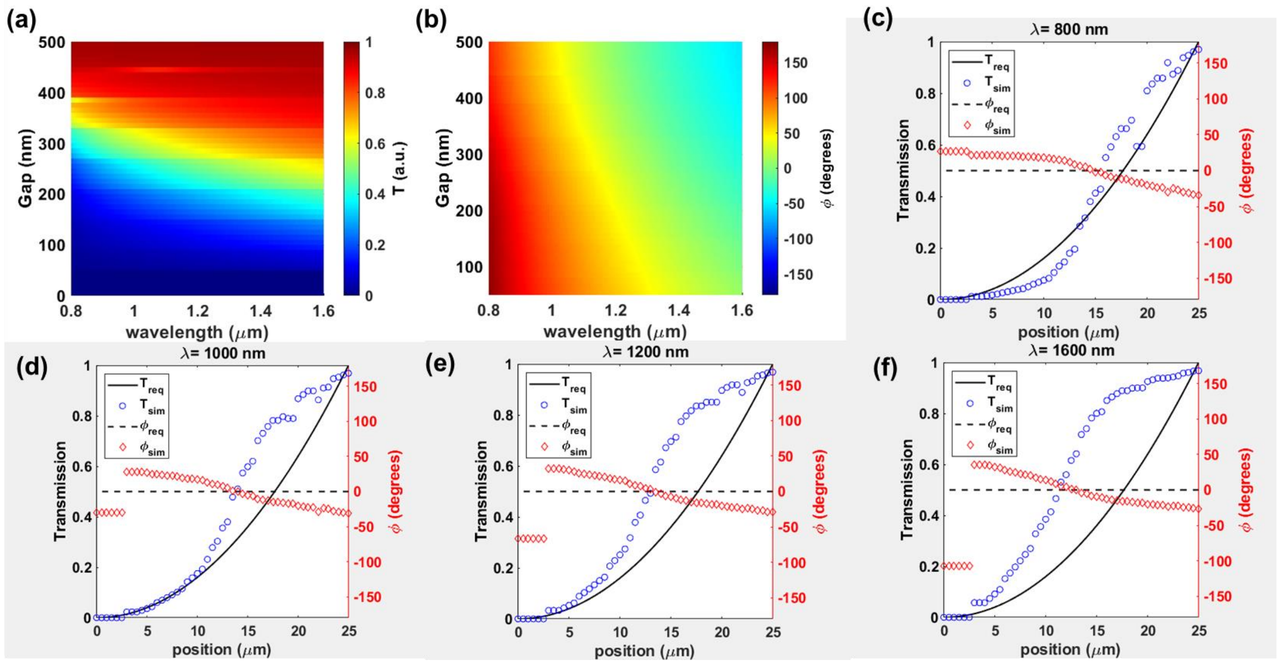

Figure S3. Unit Cell Simulations in NIR. (a) Transmission and (b) Phase response for varying gap width from 800 nm to 1600 nm wavelengths. The required versus simulated optical response of the selected set of unit cells at (c) 800 nm, (d) 1000 nm, (e) 1200 nm, and (f) 1600 nm wavelengths.

SI-3 Additional Device Simulations in Visible Range

For the input fields used as test cases in the manuscript, to span the visible wavelength range, the output of the 4f system with the metasurface as the spatial filter is numerically calculated at 400 nm and 700 nm also. The calculated output fields are shown in Figure S4(b,c) and (e,f) for the test cases; letter “N” of Northwestern University logo and the Northwestern Wildcats logo. As seen in the Figures, the metasurface successfully performs edge detection operation in 2D with unpolarized illumination 400 nm and 700 nm wavelengths.

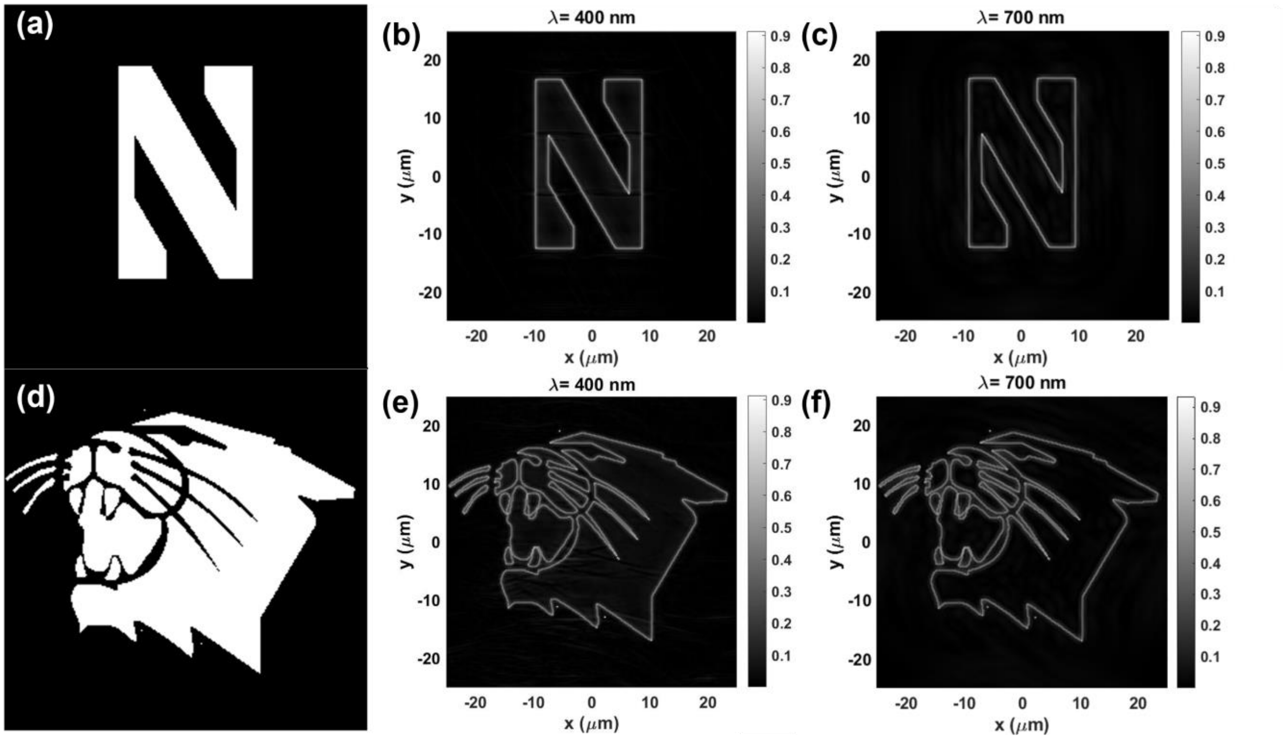

Figure S4. (a) The letter “N” of Northwestern University logo and (d) the Northwestern Wildcats logo as the input fields. and (e,f) The calculated output fields of the 4f system with the metasurface at (b,e) 400 nm and (c,f) 700 nm wavelengths, respectively.

SI-4 Device Simulations in NIR

Figure S5a-c show the distribution of the transmitted electric field amplitude and Figure S5d-f show the distribution for the phase at 800 nm, 1200 nm, and 1600 nm wavelengths, respectively. As seen in these figures, the optical response of the 3D device shows good agreement with the 2D simulations.

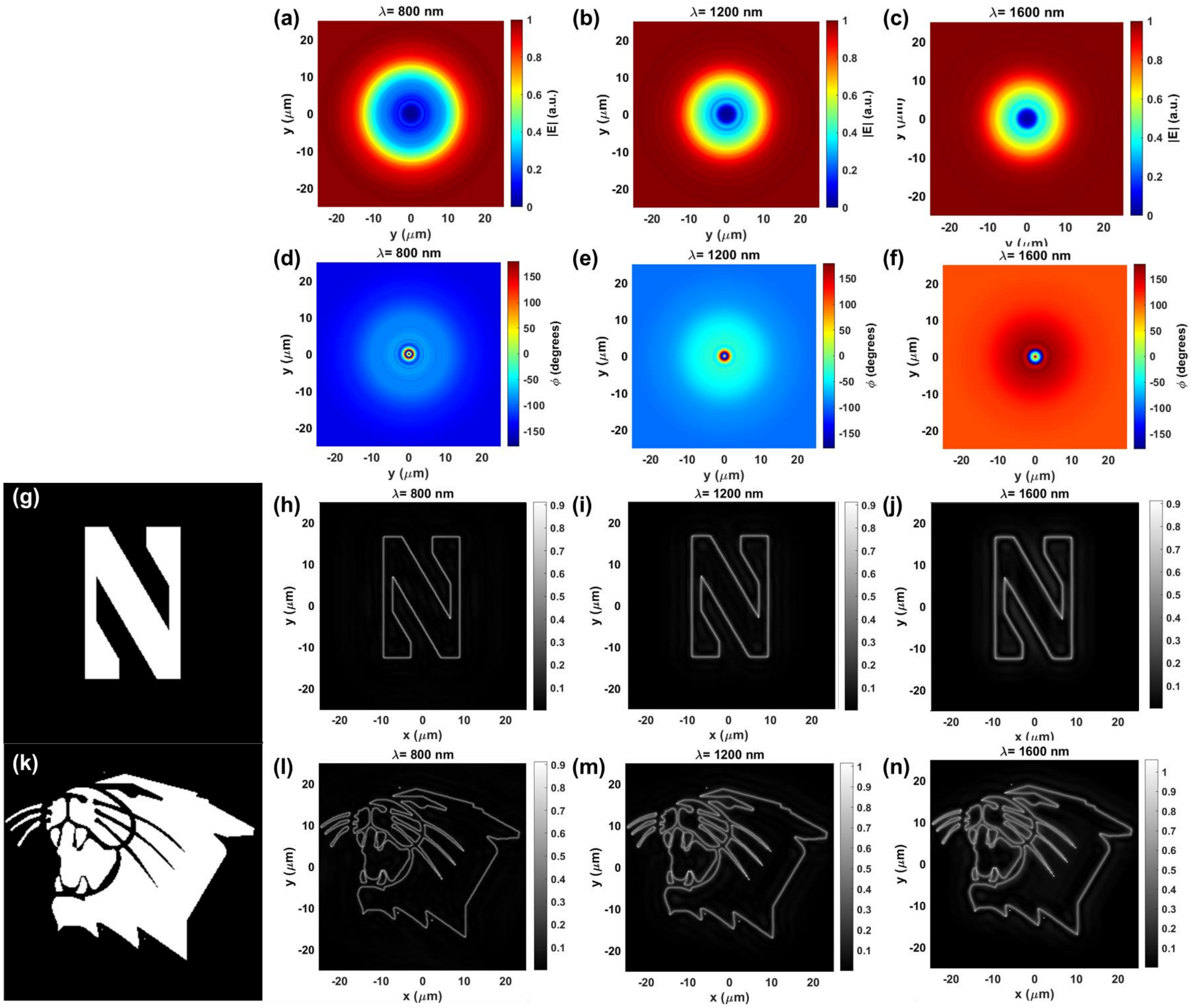

Figure S5. (a-c) The amplitude and (d-f) phase maps of the transmitted electric field at 800,1200, and 1600 nm wavelengths. (g) The letter "N" of Northwestern University logo and (k) the Northwestern Wildcats logo as the input fields. (h-j), (l-n) The calculated output fields of the 4f system with the metasurface.

For the input fields used as test cases in the manuscript, the output of the 4f system with the metasurface as the spatial filter is numerically calculated at NIR wavelengths too. The calculated output fields are shown in Figure S5(h-j) and (l-n) for the test cases; letter "N" of Northwestern University logo and the Northwestern Wildcats logo. As seen in the Figures, the metasurface successfully performs edge detection operation in 2D with unpolarized illumination 800nm,

1000nm, and 1600 nm wavelengths. Note that the clear edge detection is achieved in spite of the deviations from the target response (Figure S3 c-f) since the device maintains high pass filter operation in spatial frequency domain.

SI-5 Additional SEM Images

Figure S6 exhibits the higher magnification scanning electron microscope (SEM) images of the areas shown in Figure 3 b,c.

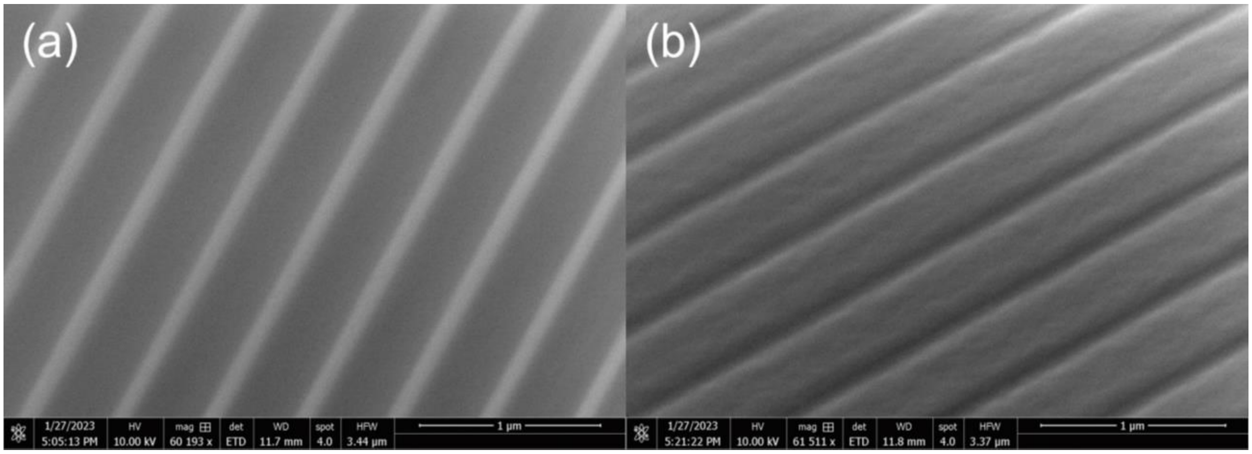

Figure S6. The SEM images of the fabricated metasurface. (a) between the center and the edge of the metasurface (area shown in Figure 3c). (b) Close to the edge of metasurface (area shown in Figure 3b).

SI-6 Spatial Transmission Profile at NIR

Figure S7 exhibits the measured spatial transmission profile in the NIR wavelengths. As seen in the Figure, the metasurface maintains high pass spatial filtering at NIR wavelengths. The measurements are in a good agreement with the simulations as seen in the Figure S3 and Figure S7.

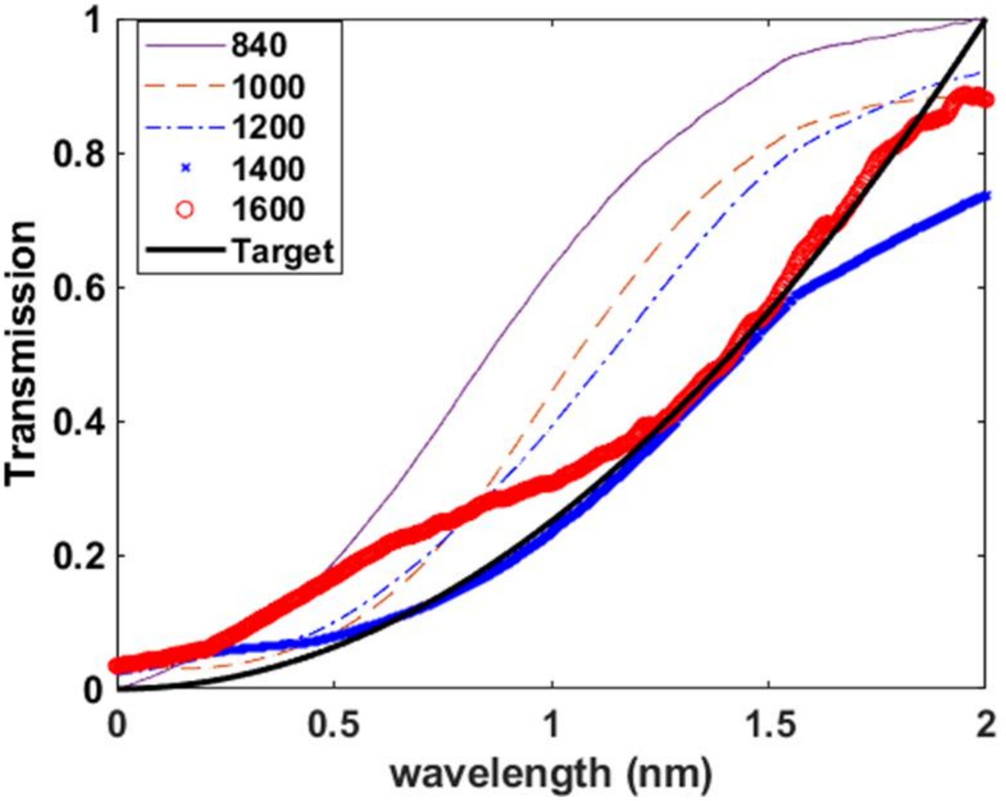

Figure S7. The measured and target spatial transmission profiles at NIR. The target (black line), 840 nm (purple line), 1000 nm (red dashed line), 1200 nm (blue dashed-dotted line), 1400 nm (blue asterisk), 1600 nm (red circle)

SI-7 White Light Experiment with Lower Input Intensity

Figure S8 shows the edge detection experiment with the incoherent unpolarized white input field. In this case, the field intensity of input is lowered to avoid detector saturation. The results for the low input intensity are used to calculate performance metric discussed in the manuscript.

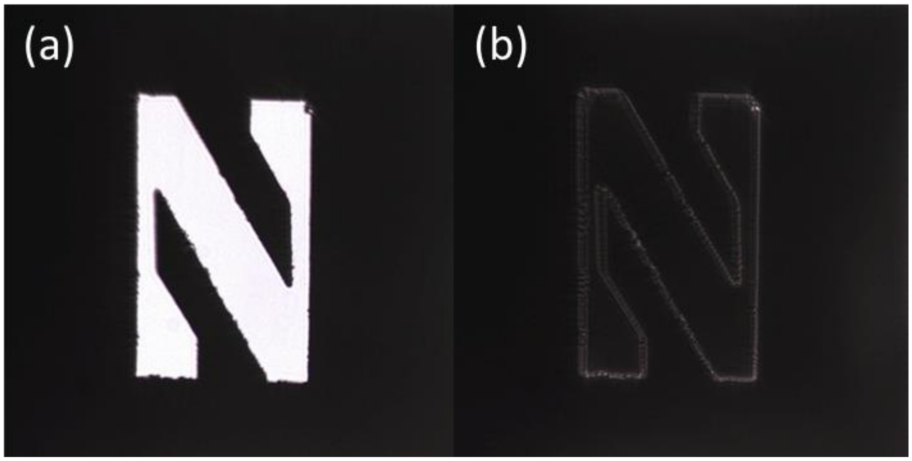

Figure S8. Edge detection experiments with Xe light bulb as the light source. (a) The reference (no metasurface) and (b) edge detected (with metasurface) images of the letter "N" of Northwestern University logo.

Supplementary References

(1) Wolfe, P. Diffraction of a Plane Wave by a Circular Disk. *J. Math. Anal. Appl.* **1979**, *67* (1), 35–57.
