# Supplementary material for: Metasurface enabled broadband all optical edge detection in visible frequencies
Source: Nat Commun. 2023 Oct 14;14:6484. doi: 10.1038/s41467-023-42271-w (PMC10576829; doi:10.1038/s41467-023-42271-w)
Supplement: Supplementary file 3 — Description of Additional Supplementary Files [file 41467_2023_42271_MOESM3_ESM.docx]

**Description of Additional Supplementary Files**

**Supplementary Video 1:** **Real-time edge detection: The letter “N” of the Northwestern University logo.** This video shows the real-time edge detection as the metasurface and the amplitude mask, the letter “N” of the Northwestern University logo, is being aligned to the same optical path. The amplitude mask is located at the object plane, the metasurface is located at the Fourier plane, and the CCD camera is located at the image plane of the 4f system as explained in the manuscript. The amplitude mask is illuminated by a Xe light bulb to create a broadband, incoherent, and unpolarized image. Initially, the metasurface is not aligned to the optical path of the 4f system. The metasurface is moved over the Fourier plane until it becomes fully aligned with the input image. The process is recorded as a video by the CCD camera.

**Supplementary Video 2: Real-time edge detection: The Northwestern Wildcats logo.** This video shows the real-time edge detection as the metasurface and the amplitude mask, the Northwestern Wildcats logo, is being aligned to the same optical path. The amplitude mask is located at the object plane, the metasurface is located at the Fourier plane, and the CCD camera is located at the image plane of the 4f system as explained in the manuscript. The amplitude mask is illuminated by a Xe light bulb to create a broadband, incoherent, and unpolarized image. Initially, the metasurface is not aligned to the optical path of the 4f system. The metasurface is moved over the Fourier plane until it becomes fully aligned with the input image. The process is recorded as a video by the CCD camera.
